# Studies on reconstruction of large skin defects following mammary tumor excision in dogs

**DOI:** 10.14202/vetworld.2017.1521-1528

**Published:** 2017-12-27

**Authors:** Sabarish Babu Malli Sadhasivan, Mohamed Shafiuzama, Mala Shammi, Ganne Venkata Sudhakar Rao, Nitin J D Souza, Hemalatha Senthilnayagam, Ravi Sundar George, P. Manoj Prabhakar

**Affiliations:** 1 Department of Veterinary Surgery and Radiology, Madras Veterinary College, Tamil Nadu Veterinary and Animal Sciences University, Chennai-600007, Tamil Nadu, India; 2 Department of Veterinary Pathology, Madras Veterinary College, Tamil Nadu Veterinary and Animal Sciences University, Chennai-600007, Tamil Nadu, India

**Keywords:** canine, histopathology, mammary tumors, reconstruction, skin fold flaps

## Abstract

**Aim:**

The main objective of the study was to describe the use of skin fold advancement flaps (SFAFs) and other reconstructive techniques for closure of large skin defects following mammary tumor excision in dogs.

**Materials and Methods:**

Twelve dogs underwent reconstruction of large ventral skin defects following mammary tumor excision with wide margins. Skin fold flaps (flank fold flap and elbow fold flap) were elevated from the flank and elbow region, respectively, and transposed and sutured onto the large ventral skin defect following mastectomy in all the dogs. In addition to the skin fold flaps, other reconstructive techniques such as undermining, walking sutures, and tension-relieving suture techniques were followed during surgery in the closure of large skin defects without skin tension and compromising limb mobility. The skin flap viability was assessed subjectively by gross observation of the flap such as color, temperature, capillary perfusion, and cosmetic appearance, and scoring (1-4) was done. Tissue samples were collected from a surgical site on days 3, 6, and 12 post-operatively for histopathological evaluation and healing status of the skin flap.

**Results:**

All the surgical wounds healed primarily, without any major complications and the skin flap remained healthy throughout the healing process post-operatively. Distal flap necrosis was noticed in one case and necrosis of skin flap between two suture lines was noticed in another case in which the necrotized distal portion healed by secondary intention after 7 days. The mean survival of subdermal plexus flap in the above cases was 98% which was a subjective evaluation based on surface area of the skin defect measured by Image’ J software and the flap dimensions. The average healing of skin flap in days was 14.91±0.86.

**Conclusion:**

The SFAFs along with other reconstructive techniques help in the reconstruction of large ventral skin defects following mastectomy in dogs without much compromising limb mobility.

## Introduction

The mammary glands in dogs are modified skin glands located bilaterally on the ventral surface of the animal from the cranial thorax to the perivulvar area which comprises of cranial thoracic, caudal thoracic, cranial abdominal, caudal abdominal, and inguinal glands from cranial to caudal region [1,2]. Surgery is the basic treatment for dogs with the most common type of mammary gland tumors (benign mixed tumor, adenoma, and adenocarcinoma), and adjuvant therapies (radiotherapy and chemotherapy) are used for inoperable tumors and also in inflammatory carcinoma [3-5]. The various surgical procedures have been described for the dogs ranging from local excision (simple mastectomy) to radical excision (radical mastectomy) and were found that there was no difference in recurrence rates and survival times following surgery [6,7]. Skin fold advancement flaps (SFAFs) were first devised as a means of closing large sternal and inguinal wounds in dogs which have four attachments such as medial and lateral attachments to the upper limb and dorsal and ventral attachments to the trunk [8,9].

In case of large mastectomy wounds created following large tumor excision in the medial thigh or sternum, unilateral or bilateral skin fold flaps (elbow fold flap or flank fold flap) may be used, respectively, to cover the extensive skin defects [10,11].

The aim of the study was to describe the use of skin fold advancement flaps (SFAFs) and other reconstructive techniques for closure of large skin defects following mammary tumor excision in dogs.

## Materials and Methods

### Ethical approval

This study does not require approval from the Ethical Committee as the study was performed in clinical cases which was presented to small animal clinical-surgical unit of Madras Veterinary College Teaching Hospital.

### Clinical cases of canine mammary tumors

The research was carried out on clinical cases of canine mammary tumors presented to the small animal surgical outpatient unit of Madras Veterinary College Teaching Hospital. Routine clinical, hematobiochemical, and radiographic examinations were carried out. A total of 12 cases of extensive mammary tumors in which the defect created after excision of tumors could not be closed conventionally were selected for the study irrespective of the breed and location of tumor mass. Fine-needle aspiration cytology (FNAC) of the mammary tumor, regional lymph nodes, and nipple aspiration fluid cytology (NAFC) was done before surgery. Thoracic radiographs were taken for each animal to assess any metastatic lesion in the lungs. The tissue samples were collected from the surgical site, post-operatively on days 3, 6, and 12 with 5 mm punch biopsy at the junction of flap with the healthy skin by local infiltration of 2% lignocaine for assessment of healing status of the skin flap. Biopsy sites were chosen randomly throughout the flap. Skin specimens were fixed in 10% buffered formalin, embedded in paraffin, and cut into 4-5 µm sections. The sections were stained with hematoxylin and eosin and examined under 100× and 400× power of compound microscope. The skin flap viability following surgery was assessed subjectively by color, temperature, capillary perfusion, and cosmetic appearance.

### Premedication and Anesthesia

The surgical site was scrubbed with povidone-iodine and prepared aseptically. The dogs were premedicated with intravenous injections of diazepam (Lori, Neon laboratories, Mumbai, India) and tramadol (Supridol, Neon laboratories, Mumbai, India) at the dose rate of 0.2 mg/kg body weight and 4 mg/kg body weight, respectively. After 15-20 min, general anesthesia was induced with propofol (Neorof, Neon laboratories, Mumbai, India) at the dose rate of 4 mg/kg body weight intravenously. The dogs were intubated immediately and general anesthesia was maintained by 1-1.5% of isoflurane (Forane, Abbott laboratories, India) with 100% oxygen with the help of a semi-open circle system.

### Surgical technique

**Figure-1:**
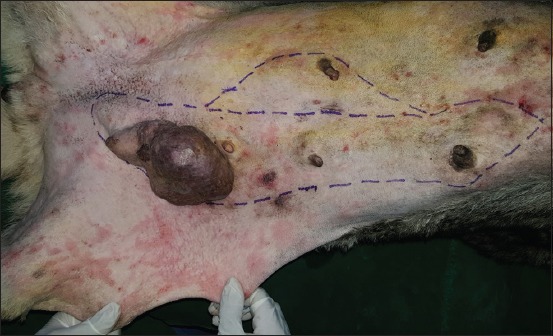
Skin marking of tumor mass and grasping of flank fold flap.

**Figure-2:**
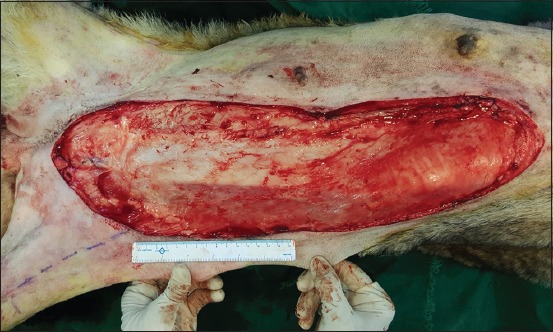
Wide resection of tumor mass with 2cm margins on all sides.

**Figure-3:**
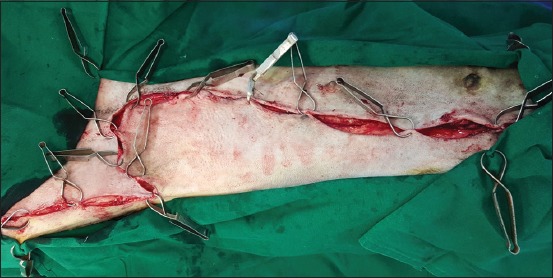
Elevation and transposition of flank fold flap onto the defect.

**Figure-4:**
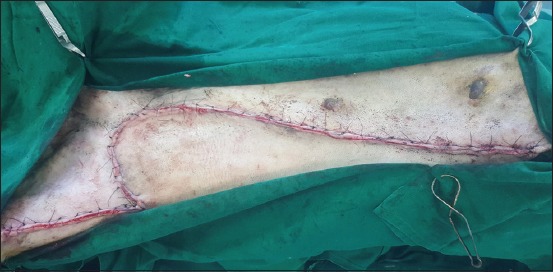
Suturing of flap onto the defect with polyamide 1-0 sutures in mattress pattern.

**Regional mastectomy** was performed in five cases, where two or more mammary glands involved as a unit with sentinel lymph node. After aseptic preparation and draping of the surgical site, 2 cm margins were marked surrounding the tumor mass using sterile skin markers (Figure-1). An elliptical skin incision was made around the base of the tumor mass over the pre-drawn margin. Careful blunt dissection was made to free the tumor from the surrounding tissue, abdominal muscles, and fascia. The tumor mass was excised completely along with the tumor capsule without leaving any tumor tissue behind. Care was taken to use separate surgical instruments for the tumor mass excision and reconstruction of skin fold flap to avoid seeding of tumor cells. Loose skin in the flank region was grasped with the hand to determine the extent of flank fold that can be harvested (Figure-2). The outline of the flank fold or elbow fold flap was marked with a sterile skin marker. Symmetrical medial and lateral incisions were made and connected with crescentic incision made proximal to elbow or stifle. The flap was undermined in a stepwise fashion, elevated from triceps or quadriceps, transposed and sutured into the defect (Figure-3). Corrugated drain sheet was placed, and it exited through the dependent part of the body for drainage of tissue fluids and to prevent seroma formation. The subcutaneous tissues were closed in continuous suture pattern with polyglycolic acid size 1-0. The skin was sutured with monofilament polyamide suture size 1-0 in mattress pattern (Figure-4).

**Figure-5:**
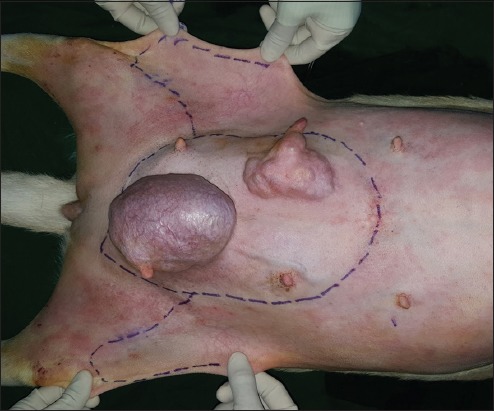
Skin marking for bilateral caudal regional mastectomy.

**Figure-6:**
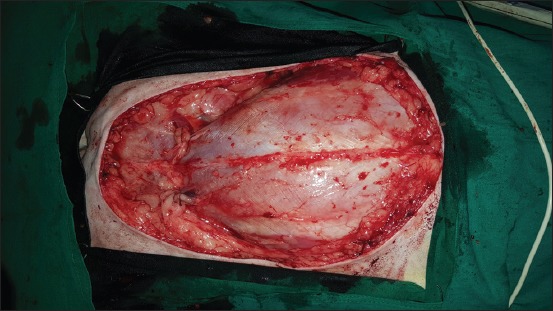
Excision of caudal two pairs of mammary glands with sentinel lymph nodes.

**Figure-7:**
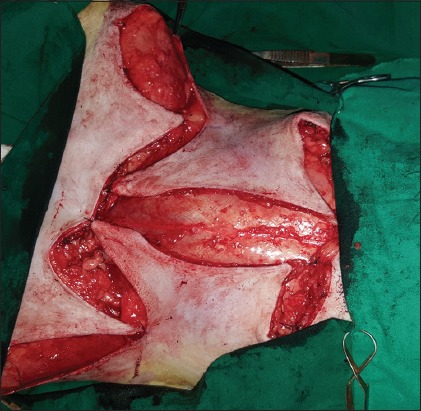
Elevation and transposition of bilateral flank fold flaps onto the defect.

**Figure-8:**
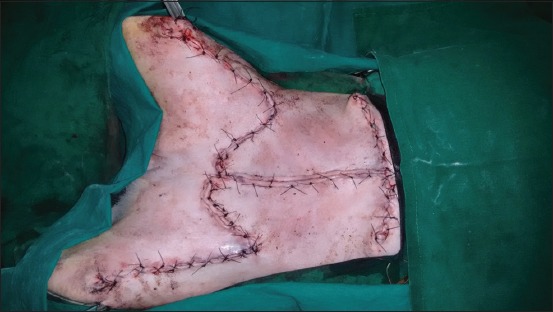
Suturing of flank fold flap with polyamide 1-0 sutures in mattress pattern.

**Bilateral caudal regional mastectomy** was performed in two cases, where tumor mass was located in the inguinal and caudal abdominal mammary gland contralaterally (Figure-5). The caudal four mammary glands along with sentinel inguinal lymph nodes were excised with wide margins (Figure-6). The remaining surgical procedure followed was similar to regional mastectomy, except bilateral flank fold flaps were elevated, transposed, and sutured onto the defect in two layers (Figure-7). Undermining, walking sutures, and tension relieving techniques were followed to close the large skin defect without much tension. Tension relieving vertical mattress sutures was placed to relieve tension at the suture site (Figure-8).

**Figure-9:**
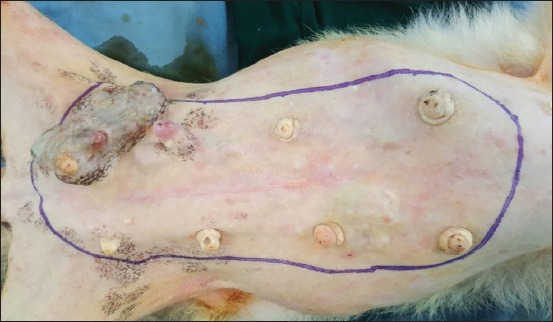
Multiple mammary masses and skin marking for tumor mass excision.

**Figure-10:**
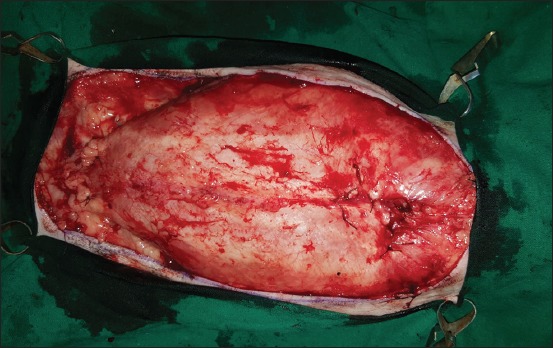
Wide margin excision of tumor mass and inguinal lymphnodes.

**Figure-11:**
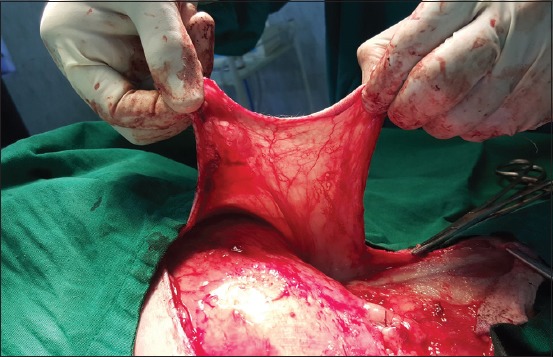
Elevation of flank fold flap from both sides of the thigh.

**Figure-12:**
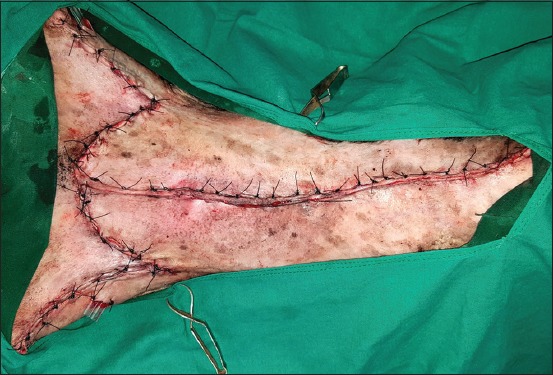
Closure of large ventral skin defect with bilateral flank fold flap.

**Bilateral mastectomy** was performed in two cases, where multiple mammary masses were present involving two or more glands on both chains and in animals with loose and pendulous mammary skin (Figure-9). The entire mammary chain was marked with sterile skin marker pen before skin incision. The incision was made along both sides of the mammary chain with 2 cm margins of healthy tissue curving to intersect cranial to the first gland and meeting caudally near the vulva. Sharp and blunt dissection was made down of the pectoral muscle, abdominal oblique muscle, or rectus fascia. The caudal and cranial superficial epigastric arteries and veins were isolated and double ligated. The superficial inguinal lymph node contained within the fat underlying the inguinal mammary tissue was excised and sent for histopathological examination. The entire mammary chain along with associated lymph nodes was removed as a single unit (Figure-10). Bilateral flank fold flap and elbow fold flap were undermined in a stepwise fashion and elevated from triceps and quadriceps region and transposed onto the defect and sutured in a routine manner(Figure-11). Walking sutures were placed between deep dermal layer and fascia on either side to obliterate dead space and distribute tensile forces throughout wound surface(Figure-12). The tumor samples were collected in 10% formalin and sent for histopathological examination.

**Unilateral mastectomy** was performed in three cases in which the entire chain of mammary glands was excised with wide margins along with lymph nodes. The large skin defect following excision of tumor mass was reconstructed with flank fold flap and elbow fold flap of that particular side (either right or left).

### Post-operative care

All the dogs were admitted in the small animal clinic-inpatient unit for 7 days for post-operative care. The surgical wound was cleaned and dressed with sterile saline, povidone-iodine, and antiseptic ointment was applied on alternate days for 7-10 days. Soft bandaging was done to promote immobilization of the skin flap. Amoxycillin-clavulanic acid at 20 mg/kg (Clavam, GlaxoSmithKline Pharma Ltd, Mumbai, India) and carprofen at 4 mg/kg (Carodyl, Savavet Pharma, India) was given orally for 7 days. The drain tube was removed on day 3 post-operatively. A punch biopsy was done under local anesthesia with 2% lignocaine, and tissue samples were collected from the surgical wound site on days 3, 6, and 12 post-operatively for evaluation of healing status of the skin flap. Supportive therapy with hematinics and Vitamin B-complex was given for speedy recovery of the patient. Skin sutures were removed on the 10^th^ post-operative day.

### Statistical analysis

Statistical analysis of the hematological and serum biochemical parameters were analyzed using paired t-test as summarized in Tables-1.1 and 1.2, and subjective evaluation of skin flap was analyzed using Kruskal–Wallis test. Both the tests were done using SPSS (Statistical Package for the Social Sciences) software.

**Table-1.1:** Statistical analysis of hematological parameters (paired ttest).

Parameter	Mean±SE		“t” value	“p” value

	Preoperatively	Postoperatively (10*^th^* day)		
Hb (g/dl)	10.14±0.61	10.54±0.26	1.01^NS^	0.33
PCV (%)	29.20±1.76	31.31±0.88	1.65^NS^	0.13
TEC (10^6^/ml)	5.41±0.33	5.37±0.22	0.25^NS^	0.81
TLC (10^3^/ml)	19.07±3.01	13.74±1.57	1.88^NS^	0.09
DC (%)				
N	79.41±1.12	77.50±1.28	2.80*	0.02
L	15.16±1.17	13.50±0.98	5.86**	0.0001
M	3.33±0.25	2.83±0.36	1.00^NS^	0.33
E	1.91±0.22	1.42±0.22	1.48^NS^	0.16

Hb=Hemoglobin, PCV=Packed cell volume, TEC=Total erythrocyte count, TLC=Total leukocyte count, DC=Differential count, SE=Standard error

**Table-1.2:** Statistical analysis of biochemical parameters (paired ttest).

Parameter	Mean±SE		“t” value	“p” value

	Preoperatively	Postoperatively (10^th^ day)		
BUN (mg/dl)	18.33±2.58	14.42±1.54	2.76*	0.02
Creatinine (mg/dl)	1.18±0.13	0.93±0.11	4.23**	0.001
Total protein (g/dl)	6.85±0.19	6.34±0.25	3.62**	0.004
Albumin (g/dl)	2.15±0.11	2.21±0.09	0.40^NS^	0.69
Globulin (g/dl)	4.7±0.27	4.18±0.28	2.65*	0.02
Glucose (mg/dl)	69.91±4.15	73.50±3.90	1.39^NS^	0.19

BUN=Blood urea nitrogen, NS=Not significant.

* Significant,

** Highly significant, >0.05 Not significant, 0.01≥p≤0.05 Significant, p<0.01 Highly significant

## Results

FNAC of mammary tumors in all the cases revealed adenocarcinoma in eleven cases and adenoma in one case. NAFC in all the cases revealed neoplastic acinar cells with a large number of mitotic figures. Histopathology of mammary tumors revealed cystic adenocarcinoma in four cases, mixed mammary tumor in three cases, tubular adenocarcinoma in three cases, fibroadenocarcinoma in one case, and adenoma in one case. Plain radiograph of thorax lateral and ventrodorsal view revealed no distant metastatic lesion in the lungs. The statistical analysis performed by paired t-test revealed that the mean hematological values of hemoglobin, packed cell volume, total erythrocyte count, and total leukocyte count did not reveal any significant difference (>0.05), whereas mean neutrophils count showed a significant difference (p<0.05) and lymphocyte count revealed high significant difference (p<0.01) between pre-operatively and 10^th^ post-operative day. Further, there was no significant difference in mean values of albumin and glucose (>0.05), whereas a significant difference was noticed in mean BUN and globulin (p<0.05) values and a high significant difference was noticed in creatinine and total protein values (p<0.01) pre-operatively and 10^th^ post-operative day. The skin flaps were also subjectively evaluated similarly to wounds on a 4-point scale, based on four criteria; color, warmthness, capillary perfusion, and cosmetic appearance. Scoring was done from 1 to 4. Evaluations were done during dressing changes on days 2, 4, and 6 post-operatively. Subjective evaluation of skin flap assessed by Kruskal–Wallis test revealed a significant difference (p<0.05) in the color and capillary perfusion scores, whereas there was no significant difference (>0.05) in the temperature and cosmetic appearance scores on days 2, 4, and 6 post-operatively. Histological evaluation of the skin flap post-operatively in all the cases revealed inflammatory phase with infiltration of neutrophils, hemorrhage, scab formation on the day 3 followed by proliferation of fibroblast, collagen fibers on day 6 and maturation of collagen fibers, and epithelialization on day 12, respectively, as stated by Gal *et al*. The mean survival of subdermal plexus flap in the above cases was 98% which was subjective evaluation based on surface area of the skin defect measured by Image’ J software and the flap dimensions, and the average healing of skin flap in days was 14.91±0.86. The summary of the size of the tumor, surgical technique performed, and flap healing is mentioned in the Table-2.

**Table-2:** Location of mammary tumor, reconstructive surgical technique performed, and flap healing in all the cases

Case No.	Tumor size (cm)	Location of tumor					Surgical technique performed	Reconstructive technique performed	Flap healing	
	
		CrT	CdT	CrA	CdA	Ing			Healing (days)	Flap survival (%)
1.	6	R	R	-	-	-	Regional mastectomy	Elbow fold flap, single pedicle advancement flap	12	98
2.	12	-	R	R, L	R, L	R	Unilateral mastectomy	Flank fold flap, tension relieving suture pattern	14	100
3.	14	-	R	R	R	R	Unilateral mastectomy	Flank fold flap	16	100
4.	10.8	-	-	R	R	R	Regional mastectomy	Flank fold flap	20	80
5.	7.8	-	-	-	R	R	Regional mastectomy	Flank fold flap	13	100
6.	10.6	-	-	-	R, L	R	Regional mastectomy	Flank fold flap, waking sutures, Tension relieving techniques	18	98
7.	7.02	-	L	L	L	L	Unilateral mastectomy	Flank fold flap, cranial superficial epigastric axial pattern flap	15	100
8.	6.8	L, R	L, R	L, R	L, R	R	Bilateral mastectomy (single stage)	Bilateral flank fold flap, tension relieving suture pattern	17	100
9.	10.3	-	-	-	R	L	Bilateral caudal regional mastectomy	Bilateral flank fold flap, walking sutures, tension relieving suture pattern	16	100
10.	18	-	L	L	L	L	Regional mastectomy	Flank fold flap	11	100
11.	6.79	-	-	-	L, R	L, R	Bilateral caudal regional mastectomy	Bilateral flank fold flap, walking sutures	17	100
12.	7.74	-	L, R	L, R	L, R	L, R	Bilateral mastectomy (single stage)	Bilateral flank fold flap	10	100
Mean±SE	9.82±1.03								14.91±0.86	98±1.65

CrT=Cranial thoracic, CdT=Caudal thoracic, CrA=Cranial abdominal, CdA=Caudal abdominal, Ing=Inguinal, L=Left, R=Right, SE=Standard error

## Discussion

The mobile skin of dogs and cats favors transposition of large pedicle or free grafts for the closure of large lumbosacral wounds and sternal and inguinal wounds [12].

In case of a large mastectomy, wounds created following large tumor mass excision in the medial thigh or sternum region, bilateral or unilateral skin fold flaps may be used, respectively, to cover the wound defects as stated by Sorenmo *et al*. [13]. The skin tension lines and pliability are important when considering a donor site to ensure that the donor site is amenable to primary closure [14,15]. In all the above cases, the skin pliability of the donor site was assessed by manual stretching of the skin in the elbow or flank region before surgery. After the flap dimensions have been established, a felt-tip pen could be used to outline the flap boundaries, providing a guide for skin incision. The flaps created should be at least 20% larger than the defect to avoid excess tension on the repair edges [16,17]. Furthermore, when creating flaps, the base should be slightly wider and the flap length should be longer than the defect, to prevent the production of excessive tension during closure [18]. The exact measurement of the defect following excision of tumor mass was made in all the cases and the flap dimensions were measured so that the recipient and donor site was opposed without tension.

The skin flap viability following surgery was assessed subjectively by color, warmthness, capillary perfusion, and cosmetic appearance as stated by Pavletic [19] and Hunt *et al*. [20]. According to Medrado *et al*. [21] and Milgram *et al*. [22], the most significant histological changes occurred during the 1^st^week of wound healing post-operatively. In the present study also, a similar histological change in skin flap was noticed as early as 6 days of flap healing. Gal *et al*. [23] stated that histological evaluation of skin wound post-operatively revealed inflammatory phase during first 3 days with infiltration of neutrophils and macrophages, whereas proliferative phase started on the 1^st^day of healing with the peak between 5 and 6 days. These findings were in accordance with the present study in all the cases, except in case 4, wherein the inflammatory phase prolonged beyond day 6 of the flap healing as there was ischemic necrosis of the distal end of the flap. Furthermore, in case 6, the proliferative phase was delayed due to infection of the flap and partial necrosis between the two suture lines.

The most common complications encountered with the above reconstructive technique was seroma in two cases, dehiscence in two cases, partial necrosis in one case, posterior limb edema in five cases, subcutaneous emphysema in one case, and hematoma in one case. Wound healing was delayed in one case where there was partial necrosis of the skin flap. In all the other cases, wound healing occurred in 10-12 days. The risk of complications can be decreased using the good surgical technique, frequent bandaging, exercise restriction, and planning appropriately before performing surgery [24,25].

The mean survival of subdermal plexus flap in the above cases was 98% which was measured subjectively as per observations of Suyoung *et al*. [26] and Remedios and Fowler [27] who reported >50% survival for subdermal plexus flaps, and the average healing of skin flap in days was 14.91±0.86.

Recurrence of new mammary tumors in the ipsilat­eral mammary chain is common following regional mastectomy in dogs as stated by Stratmann *et al*. [28] and Shafiee *et al*. [29], and hence, the authors recommend radical mastectomy rather than regional mastectomy to prevent recurrence [30].

## Conclusion

The outcome of skin flaps in all the cases was good, but small variations in hair growth pattern were noticed which was acceptable to owners. The SFAFs along with other reconstructive technique help in the closure of large ventral skin defects without much-compromising limb mobility, following mastectomy in dogs. All the cases were followed up for 1 year, and no recurrence of the tumor mass was noticed in the ipsilateral or contralateral mammary chain.

## Authors’ Contributions

SBMS designed the research work, got approval, and performed surgery; MS guided in surgery, approval of the research work, and statistical analysis of the research data; MS contributed in pre-operative patient assessment, diagnostic radiology, and article review; GVSR contributed in sectioning of tissue samples and histopathological examination of the tissue slides; NJDS assisted in pre-operative patient preparation, estimation of flap dimension, flap planning, and also in reconstructive surgery; HS performed histopathological examination of the tumors mass, skin tissue samples, and interpretation of results; RSG contributed in approval of research work and assisted in performing surgery; PMP assisted in surgery, post-operative care of the patient, and skin biopsy. All authors read and approved the final manuscript.
